# Gaze-independent ERP-BCIs: augmenting performance through location-congruent bimodal stimuli

**DOI:** 10.3389/fnsys.2014.00143

**Published:** 2014-09-08

**Authors:** Marieke E. Thurlings, Anne-Marie Brouwer, Jan B. F. Van Erp, Peter Werkhoven

**Affiliations:** ^1^Information and Computing Sciences, Utrecht UniversityUtrecht, Netherlands; ^2^Perceptual and Cognitive Systems, TNOSoesterberg, Netherlands

**Keywords:** BCI, ERP, gaze-independent, bimodal, tactile, multisensory, location-congruency, selective attention

## Abstract

Gaze-independent event-related potential (ERP) based brain-computer interfaces (BCIs) yield relatively low BCI performance and traditionally employ unimodal stimuli. Bimodal ERP-BCIs may increase BCI performance due to multisensory integration or summation in the brain. An additional advantage of bimodal BCIs may be that the user can choose which modality or modalities to attend to. We studied bimodal, visual-tactile, gaze-independent BCIs and investigated whether or not ERP components’ tAUCs and subsequent classification accuracies are increased for (1) bimodal vs. unimodal stimuli; (2) location-congruent vs. location-incongruent bimodal stimuli; and (3) attending to both modalities vs. to either one modality. We observed an enhanced bimodal (compared to unimodal) P300 tAUC, which appeared to be positively affected by location-congruency (*p* = 0.056) and resulted in higher classification accuracies. Attending either to one or to both modalities of the bimodal location-congruent stimuli resulted in differences between ERP components, but not in classification performance. We conclude that location-congruent bimodal stimuli improve ERP-BCIs, and offer the user the possibility to switch the attended modality without losing performance.

## Introduction

Event-related potential (ERP) based brain-computer interfaces (BCIs) can be used to actively and voluntarily control a system, e.g., for communication (Farwell and Donchin, 1988) or navigation (Bell et al., 2008; Thurlings et al., 2010). ERP-BCIs make use of stimuli that correspond to control options (e.g., “left” or “right”). The user can select an option by attending to the corresponding stimulus (target) while ignoring other stimuli (nontargets). Stimulus-locked brain responses (ERPs) differ between the attended targets and ignored nontargets.

Most ERP-BCIs employ visual stimuli and require the user to gaze at the target stimulus, i.e., such a BCI is *gaze-dependent*. When the user does not directly gaze at the target but only covertly attends to it, the high-level endogenous ERP components but not the low-level perceptual ERP-components differ from those of nontargets. This results in a reduced BCI performance in terms of classification accuracy (and hence bitrate) (Brunner et al., 2010; Treder and Blankertz, 2010). BCIs for which users do not have to gaze at the stimuli or to shift focus (alter viewing direction) in order to control it are called *gaze-independent*. An example is the Hex-o-Spell of Treder and Blankertz (2010). The importance of developing BCIs independent of the ability to shift focus has been expressed in studies investigating the rapid serial visual presentation paradigm (Orhan et al., 2012; Acqualagna and Blankertz, 2013). Yet, in that paradigm participants are required to directly focus at the visual stimuli.

When users cannot reliably direct their gaze, or when other tasks interfere with gaze, stimuli can also be presented in other modalities like the auditory modality (Schreuder et al., 2010, 2011; Höhne et al., 2011). In application domains such as driving and gaming, BCIs must be gaze-independent as gaze is required for control and navigation tasks and the visual (and auditory) channel is already heavily loaded (Van Erp and Van Veen, 2004). The tactile channel has also been suggested as a viable alternative for these situations, and Brouwer and Van Erp (2010) demonstrated the feasibility of employing tactile stimuli around the waist in a tactile ERP-BCI. The natural correspondence of tactile stimuli around the waist with navigation directions (Van Erp, 2005) makes a tactile ERP-BCI especially interesting for navigation applications.

BCI performance of tactile ERP-BCIs (Brouwer et al., 2010; Thurlings et al., 2012a,b) is generally lower than that of gaze-dependent BCIs (Thurlings et al., 2012a,b). In addition, when a BCI is used as a control device in the context of a dual-task, for example to navigate in a game, BCI performance is even lower than in BCI-only tasks (Thurlings et al., 2013). Therefore, in order to achieve effective use of BCI outside the lab, it is highly important to increase BCI performance of gaze-independent BCIs.

This study focusses on potential benefits regarding brain activity resulting from stimulus presentation in multiple sensory modalities, using a gaze-independent setup, and addresses three main research questions which are introduced in the next sections.

### The bimodal ERP-BCI

The processing and integration of multisensory stimuli is likely to cause additional neuronal activity (Ernst and Bülthoff, 2004; Driver and Noesselt, 2008; Stein and Stanford, 2008). Integration may take place at perceptual stages (Molholm et al., 2002; Philippi et al., 2008), higher cognitive stages (Schröger and Widmann, 1998), and/or during motor preparation and execution (Giray and Ulrich, 1993). Bimodal stimuli generally yield faster behavioral responses and more accurate perceptual discrimination (Gondan et al., 2005; Teder-Sälejärvi et al., 2005; Philippi et al., 2008).

Multisensory integration has extensively been investigated in cognitive science, but has barely received attention in the field of BCI. In a recent BCI-study, we showed that an additional (early) ERP component was present when participants were presented with and attended to stimuli in two modalities rather than one, due to multisensory interaction (Thurlings et al., 2012a). To the best of our knowledge, only two other BCI-related studies investigated bimodal stimuli: visual-tactile stimuli (Brouwer et al., 2010), and audio-visual stimuli (Belitski et al., 2011). In both studies the authors reported increased classification accuracies (i.e., the percentage of correctly classified target responses) for bimodal compared to unimodal conditions, which is in line with the trend we reported in Thurlings et al. (2012a).

Multisensory or bimodal ERP-BCIs can be regarded as a type of hybrid BCIs (Pfurtscheller et al., 2010a). Hybrid BCIs are BCIs that “can either process their inputs simultaneously, or operate two systems sequentially”. By allowing the processing of two inputs simultaneously, the second input could improve the classification accuracy of the first BCI (Pfurtscheller et al., 2010a; Yin et al., 2013).

As motivated in the beginning of the introduction, we are interested in multisensory BCIs, as a way to potentially increase BCI performance of (traditional) unimodal ERP-BCIs, in particularly the gaze-independent variants. From the three above mentioned bimodal studies, only Brouwer and Van Erp (2010) used a gaze-independent setup with visual-tactile stimuli. However in that study the effects on target and non-target responses of endogenous (voluntary) attention and exogenous (stimulus driven) attention were confound. Both endogenous and exogenous attention can affect ERP components (Woldorff and Hillyard, 1991), but only endogenous attention is relevant for BCI operation. Thus, the question whether or not gaze-independent ERP-BCIs benefit from bimodal stimulus presentation remains unanswered.

Multisensory integration has been shown to start as early as 80–120 ms after stimulus onset for visual-tactile stimuli (Sambo and Forster, 2009), but is modulated by endogenous attention at different stages of processing (Talsma and Woldorff, 2005). As reported in Thurlings et al. (2012a), positive effects of bimodal stimulus attending have been shown on an early stage of processing, i.e., early negative activity (N1) in the difference ERP (target minus nontarget ERP) was stronger for the bimodal compared to the unimodal conditions. However, we observed negative effects of bimodal stimulus attending on a late stage of the ERP, i.e., positive late activity (P300) in the difference ERP was stronger for one of the unimodal conditions (visual) compared to the bimodal condition. We hypothesized that the latter effect was caused by the spatial relation of the two unimodal stimuli that formed a bimodal stimulus pair. More specifically, although the spatial relation unambiguously indicated which unimodal stimuli formed a pair, those stimuli were not co-located. Possibly, this affected spatial attention and top-down stimulus processing. Therefore in the present study we focus on co-located (i.e., location-congruent) bimodal stimuli, with the expectation to lose the negative effects on late ERP components.

Thus, our first research question is: Are ERP components (quantified in the topographic Area Under the Curve or tAUC; Thurlings et al., 2012a,b) and corresponding classification accuracies of a bimodal visual-tactile ERP-BCI enhanced compared to its unimodal counterparts? We hypothesize enhanced bimodal ERP tAUCs both on early and late stages of processing when employing location-congruent bimodal stimuli in a gaze-independent setup, which should result in enhanced classification accuracies.

### Effects of location-congruency on the bimodal ERP-BCI

In case we find a benefit of bimodal compared to unimodal stimulus presentation and attending, as hypothesized in the previous section, it is relevant to know whether or not that effect depends (partly) on the spatial relation within the bimodal stimulus pairs. This is important for the designing of bimodal ERP-BCIs, especially since the most straightforward design might employ location incongruent bimodal stimulus pairs as in Thurlings et al. (2012a). In that study, a display presented visual navigation information and included visual stimuli located at the possible navigation directions. Tactile stimuli were presented around the waist, corresponding with navigation directions around us. To make the spatial relation as congruent as possible in that setup, the display was oriented in the horizontal plane, to match the horizontal lay-out of the tactile stimuli (Thurlings et al., 2012b). Therefore the bimodal stimulus pairs were directional-congruent, but not location-congruent. We showed in Thurlings et al. (2012a) that location-incongruency resulted in negative effects of bimodal (compared to unimodal) stimulus attending on a late stage, while effects on the early stage were positive (see previous section).

Literature on the effects of location-congruency is not unequivocal. According to the spatial rule (Meredith and Stein, 1986), stimuli from different modalities are only integrated when stimuli are spatially co-located (or proximate). Stein et al. (1989) showed for example that the performance of animals that were trained to approach visual stimuli is improved when matched with (unattended) auditory stimuli, but only if the visual-auditory stimulus pairs were spatially co-located (or proximate). Frassinetti et al. (2002) replicated these results in humans. However, also when bimodal stimulus-pairs are not location-congruent, behavior performance has been found to be enhanced (Gondan et al., 2005; Teder-Sälejärvi et al., 2005; Philippi et al., 2008). These studies differ in tasks, but have in common that the task does not enforce selective attention to one modality (as in the studies of Meredith and Stein), but rather both modalities need to be attended. Apparently the role of the spatial relation within multisensory information and if and how it affects multisensory integration depends on the specific circumstances (We address the role of selective attention to modality in the next section). Nevertheless, also when bimodal benefits are found for location-incongruent bimodal stimuli, behavioral performance may be further improved by location-congruency (Gondan et al., 2005). Teder-Sälejärvi et al. (2005) did not observe such a behavioral benefit, but did report differences in the ERP for location-congruent and location-incongruent bimodal stimuli after 100 ms. They concluded that there are overlapping and distinct processes involved in processing of location-congruent and incongruent stimuli.

Multisensory studies typically involve a task that requires participants to distinguish targets from nontargets based on physical stimulus characteristics, instead of on (only) spatial differences such as is the case in a BCI-setup (which uses spatial selective attention). Possibly, the role of the spatial relation is larger when the task is only based on spatial discrimination. Therefore it is important to study the role of the spatial relation of bimodal stimuli in ERP-BCIs.

Our second research question is: What is the effect of location-congruent compared to location-incongruent bimodal stimuli on the ERP tAUCs and corresponding classification accuracies in an ERP-BCI? We hypothesize positive effects for location congruent bimodal stimuli at late stages (e.g., P300 tAUC) of stimulus processing, which should correspond to enhanced classification accuracies.

### Effects of selective attention to modality on the bimodal ERP-BCI

Both exogenous and endogenous attention affect the ERP (Eimer et al., 2001). When participants are presented with bimodal stimuli, but they endogenously attend to either one or both modalities, exogenous attention involved in both cases is the same (as the physical characteristics have not changed). However, the amount of attentional resources allocated endogenously for processing the stimulus information of the two modalities involved differs between these cases (Macaluso, 2010). For example, when participants are precued and (pre)attending to the visual rather than the auditory modality, audio-visual stimuli are processed differently, resulting in enhanced early activity starting around 110 ms and peaking around 150 ms (Foxe and Simpson, 2005). Talsma et al. (2007) showed that for the earliest multisensory integration effect (a superadditive effect) of audiovisual stimuli to occur, both modalities need to be attended. Nevertheless, if only a single modality was attended integration still occurred but the process appeared to start later (after 250 ms after stimulus onset) and was dependent on which modality was attended.

Users of a bimodal ERP-BCI could choose to attend to either one or both modalities, which could affect the resulting ERP and may require modality-specific trained classifiers for optimal performance. In this study, we investigated the trade-off between possibly affected classification accuracies (when a bimodal classifier trained with attending to both modalities is used also when only one modality is attended to) and the advantage of the flexibility offered to the user to choose the modality to attend to.

Our third research question is: Does, and if so how does, attending to the visual or tactile modality, or both modalities affect ERP components’ tAUCs and corresponding classification accuracies in a bimodal ERP-BCI? We hypothesize that when both modalities (as opposed to either one alone) of bimodal (location-congruent) stimuli are attended, the early stage of the bimodal ERP tAUC is enhanced. Such an enhancement of the ERP tAUC could also result in enhanced classification accuracies.

Following up on the third main research question, a sub-question is: (3a) How do these classification accuracies depend on the degree of overlap in the attended modalities of the datasets during training and classification. That is, would it be possible to switch the attended modality during use, or does the classifier then need to be retrained? We hypothesize that classification accuracies are negatively affected if the applied classifier is trained on data with a different attended modality than the data that are being classified. When attended modalities during training and classifying partly overlap (i.e., visual and bimodal, or tactile and bimodal) higher classification accuracies are expected than when they do not overlap (i.e., visual and tactile).

## Materials and methods

### Participants

Ten students voluntarily participated in this study. Participants were aged between 22 and 26 years (mean age 23.5 years). All participants were male and had normal or corrected-to-normal vision. None had previously participated in a BCI-experiment and one was left-handed. The participants signed informed consent forms.

### Task

The task was to select one of two possible control options: left or right. In this study we used the index fingers to present stimulus information, in contrast to locations around the waist (corresponding to navigation directions around us) used in our previous tactile studies. The reason is that we here focus on a gaze-independent and location-congruent setup of bimodal stimuli, and participants should be able to comfortably perceive visual and tactile information from the same location(s). Because the bimodal stimuli should in addition be located at equal distances and angles from fixation, we opted to only employ two bimodal stimuli.

The two control options were presented sequentially in random order, at the left and right index finger through a tactile actuator, an LED, or both. To select an option, participants had to attend to a target stimulus location and modality, and count the number of tactile, visual or visual–tactile activations at that location. At the beginning of each trial the current target (i.e., a combination of finger and modality) was indicated by means of a short activation of the particular target stimulus. Participants were instructed to attend to all targets (and count them internally), and ignore nontargets. Within one trial, each control option (target and nontarget) was activated 10 times.

Note that although ERP-BCIs typically make use of more than two control options (i.e., more than one nontarget), Brouwer and Van Erp (2010) have shown that the P300 is also elicited in a 2-class tactile BCI and operation is not significantly reduced compared to a 4- or 6-class BCI.

### Design

The experiment involved six conditions, named after the type of stimuli and attended modality involved. In four conditions targets had to be attended in the modalities that the stimuli of that condition were presented in (no selective attention to modality): Visual, Tactile, Bimodal, Bimodal-Incongr (i.e., short for “Incongruent”). In the Bimodal condition a control option consisted of the simultaneous activation of a visual and tactile actuator at the same finger, while for Bimodal-Incongr a visual and tactile actuator of opposite fingers were matched. In the two other conditions, only one modality had to be attended, while bimodal (location-congruent) stimuli were presented. For attending the visual or tactile modality, the conditions were named: Bimodal-Att-V and Bimodal-Att-T, respectively. The order of the conditions was counterbalanced over participants.

Each condition consisted of three sets. In each set, each of the two control options was designated as the target three times, i.e., there were six trials. Each trial consisted of 10 consecutive repetitions of the control options in random order, i.e., in each set there were 60 target and 60 nontarget activations. The data of the first two sets (the training sets) were used for the training of a classifier, which was applied to classify the data in the third set (the test set). Online BCI-feedback was given to participants in the test set about which stimulus was classified as the target. The training (but not the test) set was also used for the analysis of participants’ ERP components.

### Materials

#### General

An actuator pair, consisting of a tactile vibrator and a visual LED, was attached with Velcro to each index finger (22 degrees from a fixation cross). The target and nontarget stimuli consisted of a single pulse with a pulse duration of 187.5 ms. The interval between pulses was 437.5 ms. To indicate the designated target control option at the beginning of a trial and the classified control option for BCI-feedback at the end of a trial, a 2 s and a 1 s single pulse were presented, respectively.

#### Stimuli

*Tactile stimuli*: The tactile stimuli were presented through a vibrating element called a tactor. The tactors were custom built and consisted of a plastic case with a contact area of 1 × 2 cm containing a 160 Hz electromotor (TNO, The Netherlands, model JHJ-3, see: Van Erp et al., 2007). To prevent participants from perceiving auditory information from the tactors, they listened to pink noise via speakers during the experiment in all conditions.

*Visual stimuli*: Visual stimuli were presented through two white LEDs of 5 mm, 3.2 V.

*Bimodal stimuli*: For all bimodal conditions, except for Bimodal-Incongr, bimodal stimuli consisted of the simultaneous activation of the visual and tactile stimulus on the same index finger (location-congruent). For the Bimodal-Incongr condition, the visual stimulus of one index finger and the tactile stimulus of the other index finger were activated simultaneously.

#### EEG recording equipment

EEG was recorded from eight linked-mastoids-referenced scalp electrodes (F_z_, C_z_, P_z_, O_z_, P_3_, P_4_, PO_7_, PO_8_) that used a common forehead ground (g.Tec medical engineering, GmbH). The impedance of each electrode was below 5 kΩ, as was confirmed prior to and during the measurements. EEG data were recorded with a hardware filter (bandpass 0.1–60 Hz, notch at 50 Hz) and sampled at a frequency of 256 Hz.

### Data analysis

#### EEG preprocessing and selection

To prepare the recorded EEG for ERP-analysis, we followed similar procedures as taken in Thurlings et al. (2012a,b): selecting (non)target responses, baseline correction, threshold rejection of responses, and computation of a difference ERP. However, the data were not additionally low-pass filtered (the relatively large band was chosen because of potential multisensory effects in the 30–60 Hz band).

*Selecting (non)target responses*: For ERP-analysis, both target and nontarget responses were used when preceded by a nontarget. Responses preceded by a target were discarded (i.e., there were no (other) targets presented between −625 and 625 ms relative to (non)target onset) (see also Treder and Blankertz, 2010).

*Baseline correction*: For the selected targets and nontargets, epochs from all electrodes were extracted from −100 to 625 ms relative to stimulus onset and baseline corrected relative to the average voltage during the 100 ms preceding the stimulus onset.

*Threshold rejection of responses*: We discarded epochs from all electrodes belonging to a certain stimulus, if any epoch contained amplitude differences exceeding 100 μV, indicating movement artifacts. On average, the previous steps left us with 58.8 target epochs (with a range over participants and conditions from 35 to 70) and 54.0 nontarget epochs (with a range over participants and conditions from 32 to 67). Subsequently, the selected target and nontarget epochs were averaged per participant, per condition and per electrode.

*Difference ERP*: We subtracted the averaged clean nontarget epochs from the averaged clean target epochs for each participant, each condition and each electrode. With this step, we removed exogenous (involuntary or automatic) attention effects. Further analyses were performed regarding this difference ERP (or endogenous ERP).

#### Identifying and quantifying ERP components

To identify and quantify ERP components triggered by endogenously attended stimuli, we applied the detection method as reported in Thurlings et al. (2012a,b). Only data of the training set was used, to prevent influence of BCI feedback. We identified significant effects of attending stimuli by performing a sample-by-sample *t*-test on the difference ERP (between 0 and 625 ms relative to (non)target onset) for each electrode and condition and clustered the stable segments (i.e., in this case at least seven consecutive significant samples; see also: Guthrie and Buchwald, 1991). Clusters were considered robust if they contained segments of at least two electrodes. These robust clusters defined the topographic distribution and the interval of the endogenous ERP components, taking the beginning of the earliest segment and the ending of the latest segment in the clusters as ERP component intervals.

We quantified the endogenous ERP components by using the tAUC-value (topographic Area Under the Curve), as described in Thurlings et al. (2012a,b). The tAUC reflects the magnitude of an ERP component not only by taking the averaged amplitude and duration of the component into account but also by considering the topographic distribution.

#### Online and offline BCI performance

Classification accuracies were calculated both online and offline. Online analysis was performed using BCI2000 (Schalk et al., 2004), which made use of SWLDA (stepwise linear discriminant analysis) on epochs from 0–797 ms after stimulus onset, decimation factor 4 (i.e., 64 Hz), and other standard parameters (maximum of 60 features, *p*-values initially included and backward excluded from the model <0.1 and >0.15 respectively; see Krusienski et al., 2008). The classifier was trained using the training set for each participant and for each condition.

We investigated classification accuracies more detailed offline. Standard classification parameters are based on visual ERP-BCI research. We established potentially more appropriate parameters for bimodal BCIs, and using those parameters assessed the results of the research questions in practical use. To this end, for all conditions we executed a parameter sweep with all combinations of decimation factors (between 4 and 26), and the number of blocks of downsampled windows (between 1 block and the maximum number of blocks approaching a correspondence of 800 ms). The parameter-pair resulting in the highest overall classification accuracies (averaged over all six conditions) after 10 repetitions was selected for further analyses. Then for each condition, we calculated accuracies after each repetition and established the number of repetitions which is expected most appropriate in practical use. We considered the number of repetitions the most appropriate, when classification accuracies of 70% or higher were achieved using a minimal number of repetitions (Kubler et al., 2004; Birbaumer and Cohen, 2007; Pfurtscheller et al., 2010b).

For all conditions, classification accuracies were determined by classifying the test set using a classifier trained on the training set. Additionally, to assess what the costs on BCI performance are of switching attended modality during BCI operation, analysis was done cross-conditionally: that is, for each of the conditions Bimodal, Bimodal-Att-V, and Bimodal-Att-T, the test set was classified using a classifier trained on the test set of each of the other two conditions. From the nine resulting classes of responses, we clustered three categories: (1) trained and tested on data of the same condition (“Equal”); (2) trained and tested on data of two different conditions, but with an overlap in the attended modality (e.g., trained on Bimodal, tested on Bimodal-Att-V; attending of the visual modality is overlapping) (“Overlap”); and (3) trained and tested on data of two different conditions, but without an overlap in the attended modality (e.g., trained on Bimodal-Att-V, tested on Bimodal-Att-T (“No Overlap”). Within each category the included classes were averaged per participant.

#### Statistical analysis

ERP components’ tAUC-values and classification accuracies were statistically analyzed using Statistica 8.0 (StatSoft, Tulsa, USA). To test for normality Shapiro-Wilk tests were applied, and when normality could not be assumed the data were log-transformed. We used separate one-way repeated-measures ANOVAs to examine different subsets of data appropriate to answer each of the three research questions when comparing three conditions, and paired *t*-tests when comparing two conditions. The dependent variables were tAUCs and classification accuracies. For the three main research questions, the independent variables were: (1) Bimodality (three levels: Visual, Tactile, Bimodal); (2) Location-Congruency (two levels: Bimodal, Bimodal-Incongr); and (3) Attending Modality (three levels: Bimodal, Bimodal-Att-V, Bimodal-Att-T). For sub-question (3a) the dependent variable was classification accuracies, and the independent variable was Cross-training (three levels: Equal, Overlap, No Overlap). Tukey *post-hoc* tests were applied when appropriate.

### Procedure

After the participant was verbally instructed and had read and signed the informed consent form, we attached the visual-tactile actuator pairs on his index-fingers using Velcro. The participant was seated in a dimly lit, electromagnetically shielded room and positioned his arms on the desk in front of him. We allowed the participant to become accustomed to the stimuli, by activating them for several minutes. The participant was asked to gaze at the fixation cross in front of him on the table.

During EEG preparation, we repeated the outline of the experiment and instructed the participant to move as little as possible during stimulus presentations. Before each condition, we informed the participant about the oncoming condition. When the participant indicated to be ready to begin, we started the condition. In the test sets, online BCI feedback was given after each trial (i.e., the 10th repetition). Each condition (including two training and one test recording) took approximately 3.8 min recording time. Conditions followed each other with 1–15 min breaks in between, depending on the participant’s preferences.

## Results

First we describe the general observed results (General) considering ERP components and BCI performance for each condition. Subsequently, the effects for each of the three main research questions (regarding the effects of bimodality (The effect of Bimodality), location-congruency (The effect of Location-Congruency), and selective attention to modality (The effect of Selective Attention to Modality)) are reported both with respect to ERP components and classification accuracy. Additionally, the effect of the sub-question of the third main research (addressing the effect of Cross-training) is presented in terms of classification accuracies (Classification accuracies of Cross-training (attended modality cross classifier)).

### General

#### Endogenous ERP components

**Figure 1:**
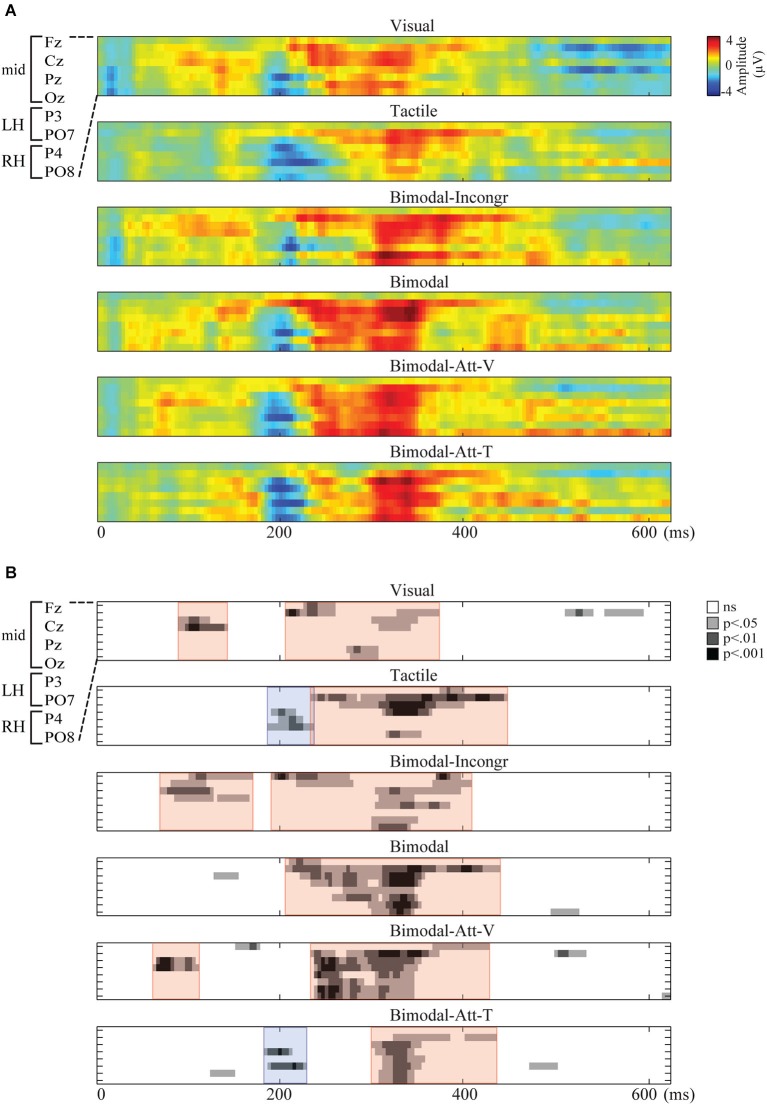
**Spatiotemporal representations of the endogenous ERP for each condition, with time (ms) on the x-axis and electrodes on the y-axis**. Electrodes from top to bottom: F_z_, C_z_, P_z_, O_z_, P_3_, P_4_, PO_7_, PO_8_. **(A)** The Grand Average of the amplitudes of the endogenous ERP (μV) for each condition. **(B)** The statistical significance of the endogenous ERP (*p*-values) resulting in stable segments, clustered in ERP components. ERP components are marked by colored overlays in red and blue for positive and negative components, respectively.

Spatiotemporal presentations of the amplitudes of the endogenous ERPs are presented in Figure 1A. For all conditions, endogenous activity was observed during one or two periods within the analyzed interval from 0 until 625 ms after stimulus onset. In Figure 1B, spatiotemporal plots show the significant stable segments. The red and blue areas in that figure indicate the polarities (positive and negative, respectively) of the clustered segments that were found to be robust and were thus identified as endogenous ERP components.

**Figure 2:**
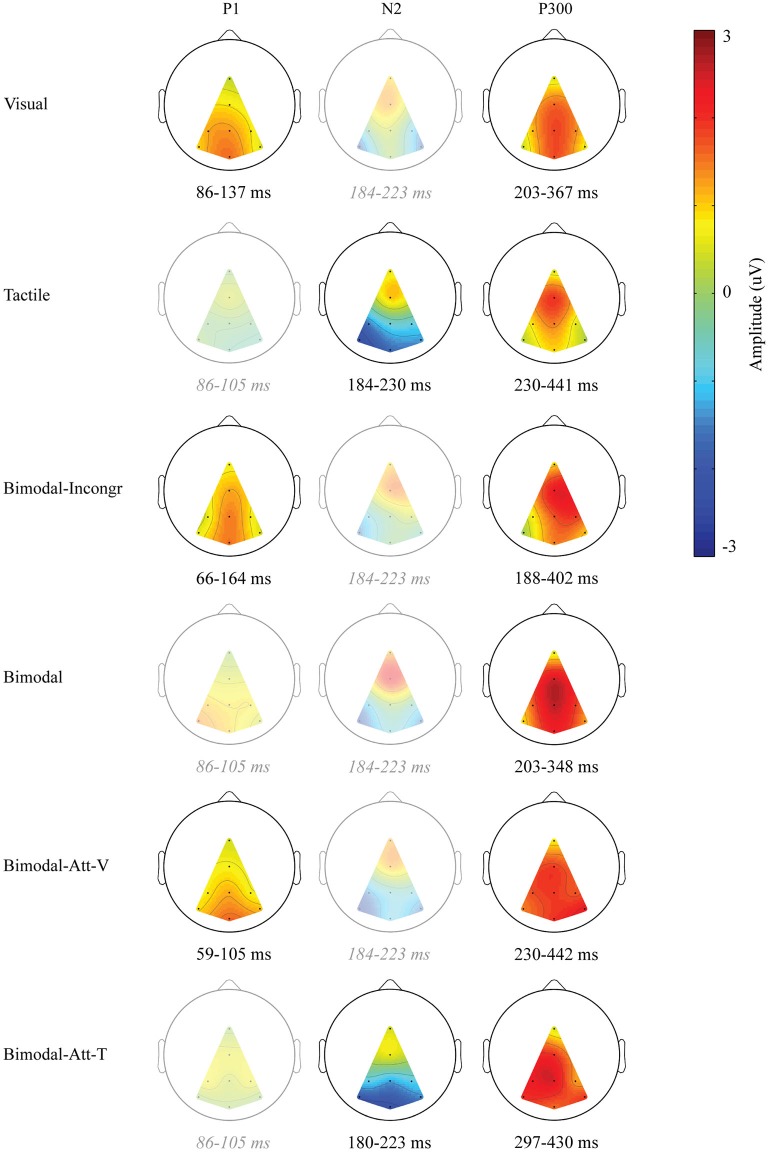
**Scalp distributions of the endogenous ERP for the identified endogenous ERP components**. Only that part of the scalp is visualized, in which electrode information could be interpolated. Amplitudes (μV) are averages calculated within each ERP component’s interval, averaged over participants. If no ERP component was identified, the overlapping interval (of the windows of the ERP component for conditions in which it was identified) was used to visualize that activity for comparison. In that case, the scalp plot is left semitransparent, and the corresponding interval is shown in gray and italics.

As apparent from Figure 1B, only one endogenous ERP component was identified in all six conditions: the P300. In Figure 2, the ERP components are visualized by means of scalp plots (averaged amplitudes of the endogenous ERP at all electrodes, within the ERP components’ intervals). The P300 amplitudes were largest in the central-parietal area, it appeared to be the strongest in the Bimodal and Bimodal-Att-V conditions, and the weakest for the Visual condition. The windows in which the P300 was detected were: 203–367 ms (Visual), 230–441 ms (Tactile), 188–402 ms (Bimodal-Incongr), 203–348 ms (Bimodal), 230–442 ms (Bimodal-Att-V), and 297–430 ms (Bimodal-Att-T) after stimulus onset.

Furthermore, early positive activity was detected and identified as P1 for the Visual, Bimodal-Att-V and Bimodal-Incongr conditions, in the windows 86–137 ms, 59–105 ms, and 66–164 ms respectively, after stimulus onset. For the Tactile and Bimodal-Att-T conditions a different early component was detected. Early negative activity was identified as an N2 in the windows 184–230 ms, and 180–223 ms respectively after stimulus onset.

**Figure 3:**
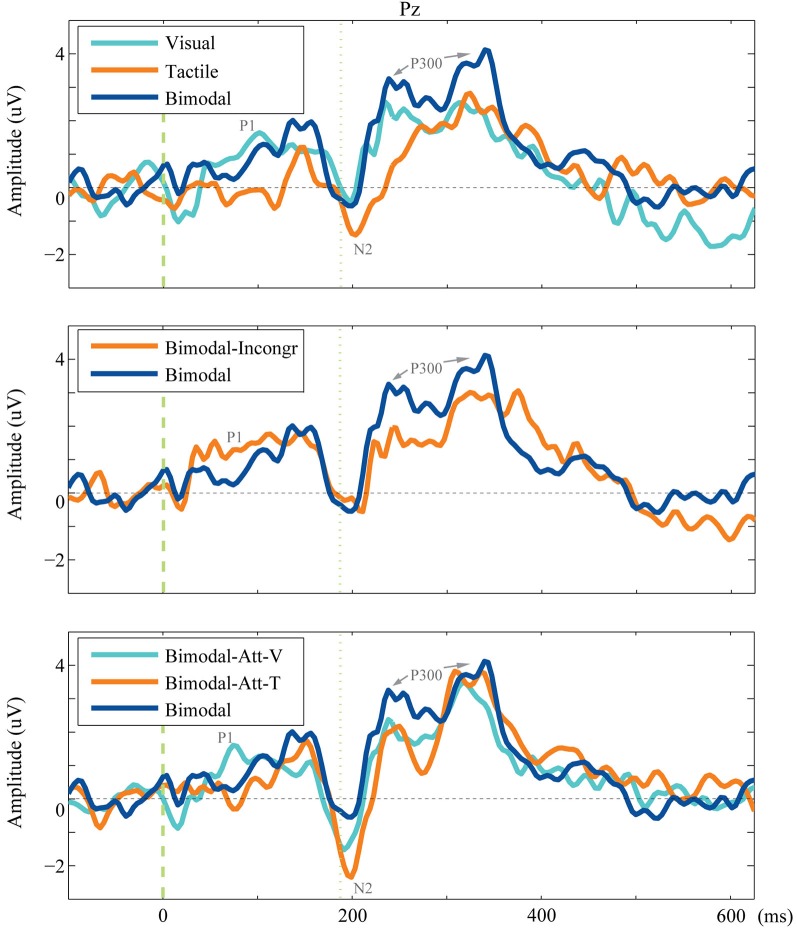
**Grand average of the endogenous ERP**. The averaged endogenous ERP is visualized for electrode P_z_ for each condition included in the comparison for each research question.

The complete ERPs are visualized for each condition for electrode P_z_ in Figure 3 (grouped per research question). The main effect of conditions on the ERP components’ tAUC-values are visualized in Figures 5A–C.

**Figure 4:**
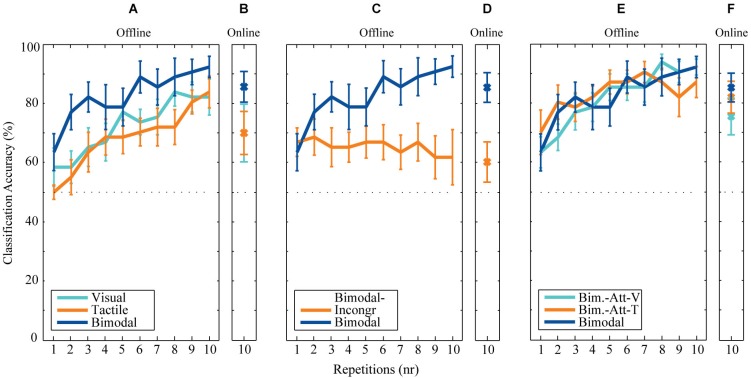
**Offline and online classification accuracies. (A)** Offline and **(B)** online classification accuracies after each repetition for the conditions involved in the analysis of the first research question (the effect of Bimodality). **(C)** Offline and **(D)** online classification accuracies after each repetition for the conditions involved in the analysis of the second research question (the effect of Location Congruency). **(E)** Offline and **(F)** online classification accuracies after each repetition for the conditions involved in the analysis of the third research question (the effect of Attending Modality).

**Figure 5:**
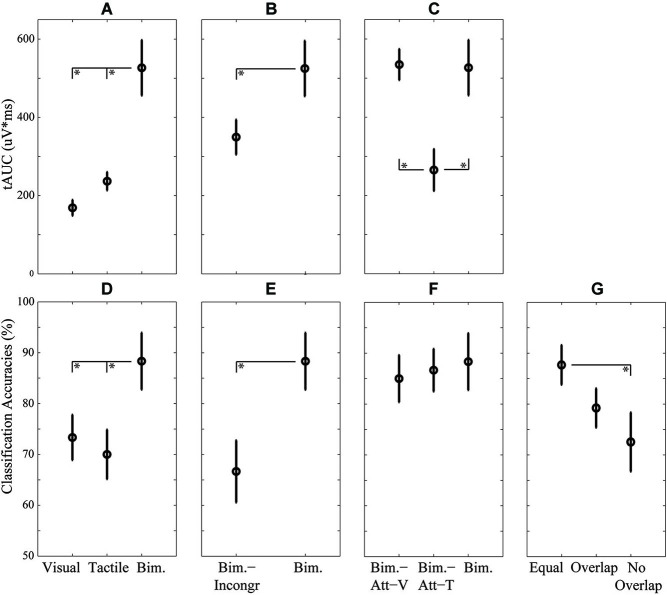
**Mean and standard errors over participants of the P300 (A–C) and classification accuracies (D–G), for each research question: the effect of modality (A,D), the effect of location-congruency (B,E), the effect of selective attention to modality (C,F), and the effect of cross-training (G)**. Condition pairs that significantly differed from each other are indicated by an asterisk (*) symbol.

#### BCI performance

A parameter-sweep was performed for combinations of decimation factors and the length of the epoch used (divided into blocks of downsampled windows). The parameter-pair of decimation factor 5 (i.e., 51.2 Hz) and epoch length of 625 ms resulted in the highest overall classification accuracies of 82.2% (SD: 11.1) over conditions. Thus, these parameters were further used for offline analysis.

Both online and offline classification accuracies are visualized in Figure 4. Overall classification accuracies (averaged over participants) are highest for all bimodal conditions employing location-congruent stimuli (i.e., Bimodal, Bimodal-Att-V, and Bimodal-Att-T). For all conditions offline classification accuracies increase with each repetition, except for the Bimodal-Incongr condition. After six repetitions, the averaged classification accuracies for five out of the six conditions exceeded the threshold of 70% necessary for effective control. For the Bimodal-Incongr condition this threshold was not reached at all. Therefore the sixth repetition is considered the most appropriate to assess effects of all research questions in a practical setting, and was used for statistical analysis.

### The effect of bimodality

#### ERP components’ tAUCs

The P300 tAUC (Table 1) was significantly affected by Bimodality (*F*_(2,18)_ = 23.93, *p* < 0.001). The P300 tAUC was larger for the Bimodal condition compared to both unimodal conditions (both *p* < 0.001), and did not differ significantly between the unimodal conditions (Figure 5A).

**Table 1:** Mean and standard errors averaged over participants of the tAUC-values (μV*ms) of all identified ERP components for each condition.

ERP Component	Visual	Tactile	Bimodal-Incongr	Bimodal	Bimodal-Att-V	Bimodal-Att-T
**P1**	37.6 (17.8)		92.3 (49.4)		36.4 (14.1)	
**N2**		56.5 (27.9)				44.4 (22.7)
**P300**	168.9 (63.5)	237.0 (73.4)	351.3 (140.9)	526.7 (223.3)	534.7 (125.4)	265.2 (168.0)

The P1 was only identified for the Visual condition (neither for Tactile nor for Bimodal) and the P1’s tAUC (Table 1) differed significantly from 0 (*t*_(9)_ = 6.69, *p* < 0.001).

The N2 was only identified for the Tactile condition (neither for Visual nor for Bimodal) and the N2’s tAUC (Table 1) differed significantly from 0 (*t*_(9)_ = 6.41, *p* < 0.001).

#### Classification accuracies

The effect of Bimodality on classification accuracies was significant (*F*_(2,18)_ = 7.30, *p* < 0.01), with higher accuracies for Bimodal compared to Visual (*p* < 0.05) and Tactile (*p* < 0.01) (Figure 5D).

### The effect of location-congruency

#### ERP components’ tAUCs

An increased P300 tAUC (Table 1) for Bimodal compared to Bimodal-Incongr approached significance (*t*_(9)_ = 2.19, *p* = 0.056) (Figure 5B).

The P1 was only identified for the Bimodal-Incongr condition (not for Bimodal) and the P1’s tAUC (Table 1) differed significantly from 0 (*t*_(9)_ = 5.91, *p* < 0.001).

#### Classification accuracies

An effect of Location-Congruency on classification accuracies was found, with higher accuracies for Bimodal compared to Bimodal-Incongr (*t*_(9)_ = 3.88, *p* < 0.01) (Figure 5E).

### The effect of selective attention to modality

#### ERP components’ tAUCs

The P300 tAUC was significantly affected by Modality (*F*_(2,18)_ = 7.50, *p* < 0.01). The P300 was stronger for the Bimodal and Bimodal-Att-V conditions compared to the Bimodal-Att-T condition (*p* < 0.05 and *p* < 0.01, respectively) (Figure 5C).

The P1 was only identified for the Bimodal-Att-V condition (neither for Bimodal-Att-T nor for Bimodal) and the P1’s tAUC (Table 1) differed significantly from 0 (*t*_(9)_ = 8.19, *p* < 0.001).

The N2 was only identified for the Bimodal-Att-T condition (neither for Bimodal-Att-V nor for Bimodal) and the N2’s tAUC (Table 1) differed significantly from 0 (*t*_(9)_ = 6.20, *p* < 0.001).

#### Classification accuracies (attended modality specific classifier)

For the attended modality specific classifier, the data used for training of the classifier and for the actual classification are recorded under the same attending-modality conditions (using bimodal location-congruent stimuli only).

No effect of Attending Modality on classification accuracies was found (Figure 5F).

#### Classification accuracies of Cross-training (attended modality cross classifier)

In this subsection the results of sub-question 3a are reported. For the attended modality cross classifier, training of the classifier occurred for each of the attending-modality conditions, and the resulting classifier was used to cross-classify the data of each of the attending-modality conditions.

Table 2 shows the results of the cross-condition classification analyses. In Figure 5G the effect of Cross-training on the clustered categories is visualized. Cross-training affected classification accuracies (*F*_(2,18)_ = 4.86, *p* < 0.05), with higher accuracies for Equal compared to No Overlap (*p* < 0.05).

**Table 2:** **Classification accuracies (averages and standard deviations) for each class of cross-conditional classification**.

Tested on	Trained on Bimodal	Bimodal-Att-V	Bimodal-Att-T
**Bimodal**	83.33 (17.66)^*^	76.67 (21.08)^∧^	80.00 (18.92)^∧^
**Bimodal-Att-V**	81.67 (12.30)^∧^	85.00 (14.59)^*^	73.33 (14.05)^#^
**Bimodal-Att-T**	76.67 (17.92)^∧^	71.67 (26.12)^#^	86.67 (13.15)^*^

Symbols *∧# indicate which classes are categorized together, with ^*^ for Equal, ^∧^ for Overlap, and ^#^ for No Overlap.

## Discussion

### The effect of bimodality

The first and main research question addressed in this study was: Are ERP components’ tAUCs and corresponding classification accuracies of a bimodal visual-tactile ERP-BCI enhanced compared to its unimodal counterparts? As we hypothesized, we found an enhanced late effect on the ERP (P300 tAUC) and corresponding enhanced classification accuracies for the location-congruent bimodal compared to the unimodal conditions, using a gaze-independent setup. In our previous bimodal work (Thurlings et al., 2012a), we did not find an enhanced bimodal P300. Instead, the bimodal P300 was even decreased compared to the visual P300. We hypothesized that effect to be a result of location-incongruent bimodal stimuli, as the P300 is affected by spatial attention (Kramer and Strayer, 1988). In this study, we showed that attending to (location-congruent) bimodal (compared to unimodal) stimuli does indeed result in an increasement of the P300 tAUC. The different findings in these two studies hint that location-congruency may indeed affect the processing of bimodal stimuli, which we will further discuss in the next section (The effect of Location-Congruency).

In contrast to our expectations, we did not find positive effects of attending to bimodal stimuli on the early stage of processing. In fact, we did not detect an early bimodal ERP component at all for location-congruent stimuli when both modalities were attended. However we did observe early ERP components for both unimodal conditions: a visual P1 and a tactile N2. Because the unimodal conditions resulted in early ERP components with opposite polarities, the lack of a bimodal early ERP component in this study may be explained by counterbalanced activity. In Thurlings et al. (2012a) we did find a bimodal early ERP component (N1), which was not detected in either of the unimodal conditions. The early ERPs of those unimodal conditions, however, appeared much more alike and already showed a slight negative drift. Also in Talsma and Woldorff (2005)—in which positive effects of audiovisual stimulus attending on early and late stages of processing are reported- the unimodal early ERPs were quite alike. The same is the case in other bimodal studies (e.g., Gondan et al., 2005; Teder-Sälejärvi et al., 2005; Philippi et al., 2008). In multisensory literature there is an on-going debate about whether or not superadded activity is elicited when multisensory integration takes place, and how integration effects can be measured (Barth et al., 1995; Gondan and Röder, 2006; Boll and Berti, 2009; Senkowski et al., 2011). Perhaps ERP summation is the driving factor behind enhanced effects of bimodal compared to unimodal ERPs in our study.

If unimodal ERP components need to be alike to elicit bimodal effects usable in BCI, it is relevant to understand why in the current study this was not the case. For the tactile condition the early ERP component (N1) resembles the tactile N2 described in Thurlings et al. (2012a), but occurred slightly (~25 ms) earlier in this study. The P1 from the visual condition also occurs ~25 ms earlier compared to the visual N2 in Thurlings et al. (2012a), but has a reversed polarity. Possibly, these visual early components do have the same generator: The polarity of the P1 can be reversed if the concerning electrode is measured in reference to for example the nose instead of linked-mastoids (Chiappa and Chiappa, 1997). Indeed in this study linked-mastoid references were used (with which a visual P1 is expected: Mangun, 1995) while in Thurlings et al. (2012a) a nose-reference was used. Not every ERP component has to be affected by such a difference: depending on the generator of a certain ERP component and the recorded electrode(s) this can affect polarity.

Attending to bimodal compared to unimodal stimuli may have increased exogenous as well as endogenous attention. Therefore the cause of the bimodality effect here could theoretically be either bottom-up, or top-down driven, or by an interaction between the two. In this study Bimodality affected the ERP positively at the late stage. Since Hopfinger and West (2006) found the P300 to be unaffected by increased exogenous attention, we think that top-down controlled endogenous attention caused the Bimodality effect. While this study does not map out the exact mechanism behind the effect, it is clear that BCI performance was much higher when congruent bimodal stimuli were used compared to unimodal stimuli. We therewith provide a way to improve performance of a gaze-independent ERP-BCI.

### The effect of location-congruency

The second research question concerned the effect of location-congruent compared to location-incongruent bimodal stimuli on the ERP components’ tAUC and corresponding classification accuracies in an ERP-BCI. As we hypothesized, we found an indication that location-congruency positively affects the late ERP component tAUC in response to bimodal stimuli (*p* = 0.056), and this trend corresponded to increased classification accuracies.

Although we only expected location-congruency to influence the late stage of the ERP, we also found a difference at the early stage: A P1 was observed for the Bimodal-Incongr condition, whereas we did not detect early ERP components for the Bimodal condition at all. This P1 resembles the P1 from the conditions in which the visual modality was relevant (Visual and Bimodal-Att-V). Therefore the occurrence of the P1 in the Bimodal-Incongr condition could be due to (stronger) attending to the visual modality. Although for that condition, participants were instructed to attend both modalities equally, the task may have been too difficult as the locations of the visual and tactile parts of the bimodal incongruent stimuli were rather far apart, and even in opposite hemifields. This could have caused participants to attend more to one of the modalities, in this case, the visual modality. The P1 seems even stronger in the Bimodal-Incongr compared to the Visual condition and compared to the Bimodal-Att-V condition, suggesting that in the Bimodal-Incongr condition participants tried to focus even more on the visual part of the stimulus to not have themselves distracted by the tactile stimulus.

BCI performance was clearly affected by location-congruency. Therefore bimodal BCIs (based on spatial attention) should be based on location-congruent bimodal stimuli for optimal performance. The performance drop caused by location-incongruent bimodal stimuli is expected to depend on the degree of incongruency.

### The effect of selective attention to modality

The third research question was does, and if so how does, attending to the visual or tactile modality, or both modalities affect ERP components’ tAUCs and corresponding classification accuracies in a bimodal ERP-BCI? We hypothesized a positive effect on the late ERP component tAUC when both modalities of bimodal (location-congruent) stimuli were attended rather than just one. Indeed attending to both modalities resulted in a stronger P300 compared to attending to the tactile modality alone, but it was equally strong as attending to the visual modality alone. Possibly, and in line with our interpretation of effects on the P1 as discussed in the previous section (processing of), the visual stimulus was dominant over (processing of) the tactile stimulus.

Selectively attending modality also had an effect on the early ERP components’ tAUCs. When the visual modality was attended in a bimodal BCI, a P1 was detected, similar as in the visual BCI. Likewise, when the tactile modality was attended in a bimodal BCI, an N2 was detected, similar as for the tactile BCI. Thus, these early ERP effects appear unrelated to multisensory interaction and solely explainable by unisensory (bottom-up) effects of stimulus processing at attended locations and within attended modalities.

#### The effect of Cross-training

BCI performance in terms of classification accuracies was equally good for the three bimodal attention conditions. This means that users can choose a preferred modality to attend to for operating a bimodal BCI when training and classifying occurs using the same attended modalities (attended modality specific classifier). We additionally assessed the effect of switching the attended modality during BCI operation by cross-classifying each one of the three bimodal attention conditions (sub-question 3a). The results indicate that when the attended modalities are different during bimodal BCI operation and training of the bimodal classifier (attended modality cross classifier), this causes a drop in BCI performance. The size of this drop depends on the degree of overlap in the attended modalities. However, even if there is no overlap, operation of the bimodal BCI is still feasible and performance is similar to that of unimodal BCIs. That means that bimodal BCIs offer the option to be used flexibly, i.e., users can switch the modality to attend to during operation.

## Conclusion

Multisensory effects can be used to enhance BCI performance (as reflected by classification accuracies) by employing bimodal stimuli. In this study we investigated bimodal effects in gaze-independent ERP-BCIs, using visual-tactile stimuli. The P300 tAUC and corresponding classification accuracies were enhanced when participants were attending to (location-congruent) bimodal vs. unimodal stimuli. Unexpectedly, we did not observe early bimodal effects for the specific condition where stimuli were location-congruent and both modalities were attended. This is possibly due to reversed polarities of early unimodal ERP components. We suggest that bimodal BCI performance may further be improved when the early unimodal ERP components are more similar, which may be achieved with different locations of the EEG reference electrode.

Furthermore, bimodal classification accuracies were improved when bimodal stimuli were location-congruent. Thus bimodal BCIs should be designed location-congruent for optimal performance.

Additionally, BCI performance was invariant for the specific modality attended, although the underlying ERP components’ tAUCs were affected. When the bimodal classifier was not trained for the specific modality attended, the drop in BCI performance depends on the degree of overlap in attended modalities between training and classifying, but was still at least as good as for the unimodal ERP-BCIs. Thus bimodal BCIs may increase BCI performance and offer more flexibility in use. This implies that for the practical use of BCIs, people who are either restricted physically or by the context of use (e.g., sensory overload of the visual channel while driving) to attend to a certain modality may benefit from using bimodal BCIs.

## Conflict of interest statement

The authors declare that the research was conducted in the absence of any commercial or financial relationships that could be construed as a potential conflict of interest.
